# (*R*)-****α****-Aminoadipic Acid: A Versatile Precursor for the Synthesis of D-Amino Acids

**DOI:** 10.1155/2013/252813

**Published:** 2013-10-10

**Authors:** Amina Sadiq, Norbert Sewald

**Affiliations:** Department of Chemistry, Bielefeld University, Universitätsstraße 25, 33615 Bielefeld, Germany

## Abstract

The ready accessibility of (*R*)-**α**-aminoadipic acid by enzymatic cleavage of cephalosporin C (CephC) in the production of 7-aminocephalosporanic acid (7-ACA) on a large scale makes it a favorable chiral pool building block for the synthesis of unusual amino acids. A route for the synthesis of C-5-alkenyl and C-6-alkylidene derivatives of (*R*)-pipecolic acid is described which utilizes (*R*)-**α**-aminoadipic acid as the enantiomerically pure starting material. Moreover, the synthesis of azido and triazolyl derivatives of (*R*)-**α**-aminoadipic acid is reported.

## 1. Introduction

*α*-Amino acids, one of the important classes of natural products, play fundamental roles in chemistry and biology. In addition to their vital roles as building block of proteins and as intermediates in metabolism, they also constitute a broad array of chiral pool building blocks and organocatalysts [1–3]. Their ready availability, low cost, and high enantiomeric purity make them valuable starting materials for the synthesis of unusual amino acids. D-Amino acids are far less abundant in nature in contrast to L-configured counterparts. In addition, D-amino acids display interesting conformational features; for example, they stabilize turn conformations in peptides [4].

*α*-Aminoadipic acid is a six-carbon analog of aspartic and glutamic acid which is found in plants and microorganisms. It is a metabolite in the principal biochemical pathway of lysine [5]. During the past decades, *α*-aminoadipic acid has received attention from chemists active in the areas of peptide chemistry, organic synthesis, biosynthesis, and neuroscience. (*R*)-*α*-Aminoadipic acid exhibits a selective antagonistic activity at the *N*-methyl-*D*-aspartate subtype of glutamate receptors [6, 7]. It has also been used in the synthesis of carbocyclic nucleoside precursors [8]. (*R*)-*α*-Aminoadipic acid **1 **is a constituent of cephalosporin C and penicillin *N*. In the pharmaceutical semisynthesis of cephalosporin derivatives, the central intermediate 7-aminocephalosporanic acid (7-ACA) is obtained by enzymatic cleavage of the fermentation product cephalosporin C (CephC) [9]. As the enantiomerically pure (*R*)-*α*-aminoadipic acid is available from this process on a large scale, we embarked on a project to explore the application of this chiral pool building block for the synthesis of (*R*)-pipecolic acid and its derivatives [10, 11].

Pipecolic acid, also known as homoproline or piperidine-2-carboxylic acid, a six-carbon natural nonproteinogenic *α*-amino acid, is an intermediate of lysine metabolism in various organisms including bacteria, yeast, fungi, and mammals. (*S*)-Pipecolic acid is an important precursor of several bioactive compounds such as synthetic peptides [12], local anesthetics [13], and potential enzyme inhibitors [14, 15] and is a component of biologically important natural products such as the immunomodulators rapamycin [16], the immunosuppressant FK506 [17], the antitumor antibiotic sandramycin [18], and the anticancer agent VX710 [19]. It also occurs in the efrapeptins and neoefrapeptins, ATPase inhibiting peptide families [20]. (*R*)-Pipecolic acid is present in the histone deacetylase inhibitors trapoxin A and apicidin [21]. These applications of pipecolic acid stimulated us to synthesize further derivatives of pipecolic acid which could be further employed in peptide modifications.

## 2. Material and Methods

All reactions were carried out in oven-dried glassware with magnetic stirrers under an argon atmosphere. THF was dried over Na/benzophenone and DCM was dried over CaH_2_. Commercially available chemicals were purchased from Sigma-Aldrich and Alfa Aesar. EtOAc and n-pentane were distilled before use. Flash chromatography was carried out using silica gel, particle size 0.035–0.070 mm. Specific rotation of synthesized compounds was recorded on a Jasco DIP-366 digital polarimeter. ^1^H NMR spectra were recorded on a 500 MHz Bruker DRX spectrometer in CDCl_3_ (unless otherwise stated) referenced relative to residual CHCl_3_ (*δ* = 7.26 ppm). Chemical shifts are reported in ppm and coupling constants in Hertz. ^13^C NMR spectra were recorded on a 500 MHz Bruker DRX (125.7 MHz) with proton decoupling. EI and CI mass spectra (including EI accurate mass measurements) were recorded using an Autospec X magnetic sector mass spectrometer with EBE geometry (Vacuum Generators, Manchester, UK) equipped with a standard EI or CI source. Samples were introduced by push rod in aluminium crucibles if not otherwise noted. Ions were accelerated by 8 kV in EI mode and 6 kV in CI mode. Accurate mass measurement experiments with ESI or MALDI were performed using a fourier transform ion cyclotron resonance mass spectrometer APEX III (Bruker Daltonik GmbH, Bremen, Germany) equipped with a 7.0 T, 160 mm bore superconducting magnet (Bruker Analytik GmbH—Magnetics, Karlsruhe, Germany), infinity cell, and interfaced with an external (nano)ESI or MALDI ion source. Nitrogen served both as the nebulizer gas and the dry gas for ESI. Nitrogen was generated by a Bruker nitrogen generator NGM 11. Argon served as a cooling gas in the infinity cell and a collision gas for MS^n^ experiments.

*Benzyl (R)-2-(Benzyloxycarbonylamino)-6-(methylsulfonyloxy)hexanoate (* ***7***). Et_3_N (1.68 mmol, 2.5 eq., 0.23 mL) and methanesulfonyl chloride (0.81 mmol, 1.2 eq., 0.06 mL) were added to a solution of **4 **(0.673 mmol, 1 eq., 250 mg) in dry CH_2_Cl_2_ (8 mL) at 0°C, and the mixture was stirred at the same temperature. The progress of the reaction was monitored by TLC. After the reaction was complete (∼45 min), it was quenched by addition of water (10 mL). The mixture was then extracted with CH_2_Cl_2_ (3 × 15 mL), and the combined organic phase was washed with brine (15 mL) and dried over anhydrous Na_2_SO_4_. Evaporation of solvent and silica gel column chromatography of the resulting residue (n-pentane/EtOAc 6 : 4) gave the corresponding mesylate **7** (270 mg, 92%) as a colorless oil.

[*α*]_D_^23^ = +23.0 (*c* = 1.5, CHCl_3_). ^1^H NMR (500 MHz, CDCl_3_): *δ* = 7.41–7.33 (m, 10H, Ar-H), 5.52 (br d, 1H, *J* = 8.3 Hz, NH), 5.24–5.15 (m, 2H, Bn-CH_2_), 5.12 (s, 2H, Bn-CH_2_), 4.39 (m, 1H, CH), 4.17–4.13 (m, 2H, CH_2_), 2.95 (s, 3H, CH_3_), 1.87 (m, 1H, CH_2_), 1.83–1.66 (m, 3H, CH_2_), 1.51–1.35 (m, 2H, CH_2_). ^13^C NMR (125.7 MHz, CDCl_3_): *δ* = 172.0 (COO), 155.9 (NCOO), 136.6 (Ar-C), 135.2 (Ar-C), 128.6 (10 Ar-CH), 69.4 (OCH_2_), 67.2 (Bn-CH_2_), 67.0 (Bn-CH_2_), 53.6 (CH), 37.2 (CH_3_), 31.9 (CH_2_), 28.5 (CH_2_), 21.2 (CH_2_). C_22_H_27_NO_7_S (449.16). HRMS (ESI-FT-ICR): *m*/*z* = 472.14057; calcd. for [C_22_H_27_NO_7_SNa]^+^: *m*/*z* = 472.14004.

*Benzyl (R)-6-Azido-2-(benzyloxycarbonylamino)hexanoate (* ***8***). NaN_3_ (1.00 mmol, 1.5 eq, 70 mg) was added to a solution of mesylate **7** (0.68 mmol, 1 eq., 300 mg) in dry DMF (8 mL). The reaction mixture was stirred at room temperature for 18 h. It was then poured into water (15 mL) and extracted with EtOAc (3 × 15 mL). The combined organic phase was washed with brine (20 mL) and dried over Na_2_SO_4_. The solvent was removed under reduced pressure and the residue thus obtained was purified by silica gel column chromatography (ether/EtOAc 7 : 3) to give **8** (202 mg, 76%) as a colorless oil.

[*α*]_D_^23^ = +30.1 (*c* = 1.5, CHCl_3_). ^1^H NMR (500 MHz, Me_3_OD): *δ* = 7.39–7.28 (m, 10H, Ar-H), 5.22–5.13 (m, 2H, Bn-CH_2_), 5.10 (s, 2H, Bn-CH_2_), 4.15 (m, 1H, CH), 3.24 (t, *J* = 6.7 Hz, 2H, CH_2_), 1.82 (m, 1H, CH_2_) 1.67 (m, 1H, CH_2_), 1.60–1.50 (m, 2H, CH_2_), 1.49–1.40 (m, 2H, CH_2_). ^13^C NMR (125.7 MHz, CDCl_3_): *δ* = 174.9 (COO), 159.7 (NCOO), 139.3 (Ar-C), 138.3 (Ar-C), 128.6 (10 Ar-CH), 68.9 (CH_2_), 68.7 (CH_2_), 56.5 (CH_2_), 53.2 (CH), 33.2 (CH_2_), 30.4 (CH_2_), 25.1 (CH_2_). C_21_H_24_N_4_O_4_ (396.18). HRMS (ESI-FT-ICR): *m*/*z* = 397.18724; calcd. for [C_21_H_24_N_4_O_4_H]^+^: *m*/*z* = 397.18703.

### 2.1. General Procedure for Copper Catalyzed Azide-Alkyne Cycloaddition (CuAAC)

Substituted alkyne (0.352 mmol, 1 eq.) was added to a solution of azide **8** (0.352 mmol, 1 eq.) in a 1 : 9 mixture of DMSO (0.5 mL) and H_2_O (4.5 mL). CuSO_4_·5H_2_O (0.088 mmol, 0.25 eq.) was added to the mixture, followed by the addition of sodium ascorbate (0.160 mmol, 0.5 eq.). The reaction mixture was vigorously stirred overnight at room temperature. After completion (monitored by TLC) the reaction was quenched by addition of water and extracted with CH_2_Cl_2_ (3 × 20 mL). The combined organic phase was washed with water and brine and concentrated in vacuo. The residue was purified by flash column chromatography (n-pentane/EtOAc 1 : 1) to give the product as colorless oil.

#### 2.1.1. Benzyl (*R*)-2-(Benzyloxycarbonylamino)-6-(4-phenyl-1*H*-1,2,3-triazol-1-yl)hexanoate (**11**)

Colorless oil, yield 100 mg (57%). [*α*]_D_^23^ = +27.3 (*c* = 1.5, CHCl_3_). ^1^H NMR (500 MHz, CDCl_3_): *δ* = 7.82–7.76 (m, 2H, Ar-H), 7.69 (s, 1H, Ar-H), 7.42–7.40 (m, 2H, Ar-H), 7.35–7.30 (m, 11H, Ar-H), 5.35 (d, *J* = 8.2 Hz, 1H, NH), 5.19–5.04 (m, 4H, Bn-CH_2_), 4.43 (m, 1H, CH), 4.31 (t, *J* = 7.1 Hz, 2H, CH_2_), 1.96–1.87 (m, 2H, CH_2_) 1.75–1.68 (m, 2H, CH_2_), 1.35 (m, 1H, CH_2_), 1.28 (m, 1H, CH_2_). ^13^C NMR (125.7 MHz, CDCl_3_): *δ* = 171.9 (COO), 155.8 (NCOO), 147.7 (C), 136.1 (Ar-C), 135.1 (Ar-C), 130.6 (3 Ar-CH), 128.8 (12 Ar-CH), 125.7 (Ar-C), 119.4 (CH), 67.3 (Bn-CH_2_), 67.0 (Bn-CH_2_), 53.4 (CH), 49.9 (CH_2_), 32.0 (CH_2_), 29.6 (CH_2_), 22.0 (CH_2_). C_29_H_30_N_4_O_4_ (498.18). HRMS (ESI-FT-ICR): *m*/*z* = 521.21663; calcd. for [C_29_H_30_NO_4_Na]^+^: *m*/*z* = 521.21593.

#### 2.1.2. Benzyl (*R*)-2-(Benzyloxycarbonylamino)-6-(4-ethoxycarbonyl-1*H*-1,2,3-triazol-1-yl)hexanoate (**12**)

Colorless oil, yield 75 mg (60%). [*α*]_D_^23^ = +21.4 (*c* = 1.5, CHCl_3_). ^1^H NMR (500 MHz, CDCl_3_): *δ* = 8.04 (s, 1H, CH), 7.39–7.31 (m, 10H, Ar-H), 5.38 (br d, 1H, *J* = 8.2 Hz, NH), 5.18–5.11 (m, 4H, Bn-CH_2_), 4.45–4.41 (m, 3H, CH_2_, CH), 4.32 (t, *J* = 7.3 Hz, 2H, CH_2_), 1.97–1.86 (m, 2H, CH_2_), 1.76–1.70 (m, 2H, CH_2_) 1.42 (t, *J* = 7.1 Hz, 3H, CH_3_), 1.37–1.26 (m, 2H, CH_2_). ^13^C NMR (125.7 MHz, CDCl_3_): *δ* = 171.8 (COO), 160.7 (COO), 155.8 (NCOO), 140.3 (C), 136.1 (Ar-C), 135.1 (Ar-C), 128.6 (10 Ar-CH), 127.2 (CH), 67.3 (Bn-CH_2_), 67.1 (Bn-CH_2_), 61.3 (COOCH_2_), 53.4 (CH), 50.4 (CH_2_), 32.0 (CH_2_), 29.4 (CH_2_), 21.9 (CH_2_), 14.3 (CH_3_). C_26_H_30_N_4_O_6_ (494.22). HRMS (EI): *m*/*z* = 494.21457; calcd. for [C_26_H_30_N_4_O_6_]^+^: *m*/*z* = 494.21599.

### 2.2. General Procedure for Ruthenium Catalyzed Azide-Alkyne Cycloaddition (RuAAC)

A mixture of azide **8** (0.252 mmol, 1 eq., 100 mg), alkyne (0.504 mmol, 2 eq.), and Cp*RuCl(PPh_3_)_2_ (0.005 mmol, 0.02 eq.) in benzene (7 mL) was stirred under reflux for 24 h. The progress of the reaction was monitored by TLC. After completion of reaction, the solvent was evaporated under vacuum, and the product was purified by silica gel chromatography (n-pentane/EtOAc 1 : 1) to give the product as colorless oil.

#### 2.2.1. (*R*)-Benzyl 2-(Benzyloxycarbonylamino)-6-(5-phenyl-1*H*-1,2,3-triazol-1-yl)hexanoate (**9**) 

Colorless oil, yield 80 mg (64%). [*α*]_D_^23^ = +27.8 (*c* = 1.5, CHCl_3_). ^1^H NMR (500 MHz, CDCl_3_): *δ* = 7.69 (s, 1H, Ar-H), 7.52–7.48 (m, 3H, Ar-H), 7.39–7.30 (m, 12H, Ar-H), 5.32 (d, *J* = 8.3 Hz, 1H, NH), 5.20–5.13 (m, 2H, Bn-CH_2_), 5.11 (s, 2H, Bn-CH_2_) 4.37 (m, 1H, CH), 4.28 (t, *J* = 7.3 Hz, 2H, CH_2_), 1.88–1.78 (m, 3H, CH_2_) 1.60 (m, 1H, CH_2_), and 1.35–1.20 (m, 2H, CH_2_). ^13^C NMR (125.7 MHz, CDCl_3_): *δ* = 171.9 (COO), 155.8 (NCOO), 137.7 (Ar-C), 136.1 (Ar-C), 135.1 (Ar-C), 133.0 (CH), 129.4 (Ar-CH), 129.1 (2 Ar-CH) 128.8 (12 Ar-CH), 127.1 (C), 67.2 (CH_2_), 67.0 (CH_2_), 53.6 (CH), 47.8 (CH_2_), 32.0 (CH_2_), 29.5 (CH_2_), 22.1 (CH_2_). C_29_H_30_N_4_O_4_ (498.23). HRMS (ESI-FT-ICR): *m*/*z* = 521.21667; calcd. for [C_29_H_30_N_4_O_4_Na]^+^: *m*/*z* = 521.21593.

#### 2.2.2. Benzyl (*R*)-2-(Benzyloxycarbonylamino)-6-(5-ethoxycarbonyl-1*H*-1,2,3-triazol-1-yl)hexanoate (**10**)

Colorless oil, yield 65 mg (52%). [*α*]_D_^23^ = +22.0 (*c* = 1.5, CHCl_3_). ^1^H NMR (500 MHz, CDCl_3_): *δ* = 8.04 (s, 1H, CH), 7.39–7.33 (m, 10H, Ar-H), 5.39 (br d, 1H, *J* = 8.3 Hz, NH), 5.22–5.14 (m, 2H, Bn-CH_2_), 5.11 (s, 2H, Bn-CH_2_), 4.45–4.30 (m, 3H, CH_2_, CH), 4.32 (t, *J* = 7.3 Hz, 2H, CH_2_), 1.96–1.86 (m, 3H, CH_2_), 1.73 (m, 1H, CH_2_) 1.39 (t, *J* = 7.1 Hz, 3H, CH_3_), 1.37–1.27 (m, 2H, CH_2_). ^13^C NMR (125.7 MHz, CDCl_3_): *δ* = 171.8 (COO), 160.7 (COO), 155.8 (NCOO), 140.3 (C), 136.1 (Ar-C), 135.1 (Ar-C), 128.6 (10 Ar-CH), 127.4 (CH), 67.3 (CH_2_), 67.1 (CH_2_), 61.3 (COOCH_2_), 53.4 (CH), 50.2 (CH_2_), 32.0 (CH_2_), 29.4 (CH_2_), 21.9 (CH_2_), 14.3 (CH_3_). C_26_H_30_N_4_O_6_ (494.22). HRMS (ESI-FT-ICR): *m*/*z* = 517.20527; calcd. for [C_21_H_23_NO_4_Na]^+^: *m*/*z* = 517.20567.

### 2.3. General Procedure for Wittig Reaction with Nonstabilized Ylids

*n*-Butyl lithium (0.1 mL, 1.6 M in hexane, 0.08 mmol) was added to a stirred solution of triphenylalkylphosphonium bromide (0.08 mmol, 1 eq.) in THF (5 mL), and the resulting mixture was stirred at room temperature for 30 min. The solution of aldehyde **3** (25 mg, 0.07 mmol) in THF (3 mL) was added dropwise to the mixture. The reaction mixture was stirred at room temperature for 1.3 h. After completion of the reaction monitored by TLC (hexane/EE, 7 : 3), it was quenched by addition of water and extracted with ether. The combined ether layers were dried and concentrated. The crude product was purified by flash chromatography on silica gel (*n*-hexane/EtOAc 7 : 3).

#### 2.3.1. Dibenzyl (*R*)-5-Vinyl-3,4-dihydropyridine-1,2(2*H*)-dicarboxylate (**13a**)

Colorless oil, yield 45.5 mg (60%) [10, 11].

#### 2.3.2. Dibenzyl (*R*)-5-(2-Methyl-1-propenyl)-3,4-dihydropyridine-1,2(2*H*)-dicarboxylate (**13b**)

Colorless oil, yield 105 mg (65%). [*α*]_D_^23^ = +33.2 (*c* = 1.5, CHCl_3_); ^1^H NMR (500 MHz, CDCl_3_, conformer mixture): *δ* = 7.49–7.29 (m, 10H, Ar-H), 6.94/6.82 (s, 1H, CH), 5.57/5.54 (s, 1H, CH), 5.30–5.11 (m, 4H, Bn-CH_2_), 4.89 (m, 1H, CH), 2.45 (m, 1H, CH_2_), 2.12 (m, 1H, CH_2_), 2.03 (m, 1H, CH_2_), 1.95 (m, 1H, CH_2_), 1.80 (s, 3H, CH_3_), 1.77/1.88 (d, *J* = 5 Hz, 3H, CH_3_). ^13^C NMR (125.7 MHz, CDCl_3_, conformer mixture): *δ* = 170.8/170.6 (COO), 153.3/153.1 (NCOO), 136.1/135.9 (Ar-C), 135.7/135.5 (Ar-C), 132.6/132.6 (C) 128.5/127.9 (10 Ar-CH), 124.4/124.4 (CH), 122.8/122.4 (CH), 116.7/116.3 (C), 67.9/67.7 (Bn-CH_2_), 66.9/66.9 (Bn-CH_2_), 53.7/53.5 (CH), 23.8/23.6 (CH_2_), 23.0/22.8 (CH_2_), 19.7/19.6 (CH_3_). C_25_H_27_NO_4_ (405.19). HRMS (EI) *m*/*z* = 405.19370; calcd. for [C_25_H_27_NO_4_]: *m*/*z* = 405.19401.

#### 2.3.3. (*R,E*)-Dibenzyl 5-(1-Butenyl)-3,4-dihydropyridine-1,2(2*H*)-dicarboxylate and (*R,Z*)-Dibenzyl 5-(1-Butenyl)-3,4-dihydropyridine-1,2(2*H*)-dicarboxylate (**13c**)

Colorless oil, yield 110 mg (68%), [*α*]_D_^23^ = +31.1 (*c* = 1.5, CHCl_3_); ^1^H NMR (500 MHz, CDCl_3_,* E*/*Z* and conformer mixture): *δ* = 7.42–7.27 (m, 10H, Ar-H), 7.02/6.91 (s, 1H, CH), 6.07/5.99_trans⁡_, 5.72/5.67_cis_ (2d, *J*_trans⁡_ = 15.7 Hz and 2d, *J*_cis_ = 11.6 Hz, 1H, CH), 5.54–5.11 (m, 5H, Bn-CH_2_, CH), 4.97 (m, 1H, CH), 2.45 (m, 1H, CH_2_), 2.31–2.03 (m, 4H, CH_2_), 1.96 (m, 1H, CH_2_), 1.05–0.95 (m, 3H, CH_3_). ^13^C NMR (125.7 MHz, CDCl_3_, conformer mixture, signals of both *E *and *Z *isomer): *δ* = 170.7/170.5 (COO), 153.2/153.0 (NCOO), 135.9/135.8 (Ar-C), 135.6/135.5 (Ar-C), 131.3/131.2 (CH) 128.5/127.9 (10 Ar-C), 127.8/127.7/127.6/127.5 (CH), 124.7/123.6/123.2/122.7 (CH)_cis&trans_, 116.6/116.2 (C), 68.0/67.8 (Bn-CH_2_), 67.0/66.9 (Bn-CH_2_), 53.7/53.4 (CH) 23.7/23.5 (CH_2_), 22.6/22.4 (CH_2_), 22.3/22.3 (CH_2_), 14.9/14.8 (CH_3_). C_24_H_27_NO_4_ (405.19). HRMS (EI) *m*/*z* = 405.19420; calcd. for C_24_H_27_NO_4_ *m*/*z* = 405.19401.

#### 2.3.4. (*R,E*)-Dibenzyl 5-(1-Propenyl)-3,4-dihydropyridine-1,2(2*H*)-dicarboxylate and (*R,Z*)-Dibenzyl 5-(1-Propenyl)-3,4-dihydropyridine-1,2(2*H*)-dicarboxylate (**13d**)

Colorless oil, yield 94 mg (61%). [*α*]_D_^23^ = +30.7 (*c* = 1.5, CHCl_3_); ^1^H NMR (500 MHz, CDCl_3_, conformer mixture, signals of both *E *and *Z *isomer): *δ* = 7.31–7.16 (m, 10H, Ar-H), 6.93/6.90/6.81/6.78 (s, 1H, CH), 5.97/5.90_trans⁡_, 5.67/5.62_cis_ (2d, *J*_trans⁡_ = 15.5 Hz and *J*_cis_ = 11.7 Hz, 1H, CH), 5.39 (m, 1H, CH), 5.19–5.00 (m, 4H, Bn-CH_2_), 4.90 (m, 1H, CH), 2.31 (m, 1H, CH_2_), 2.12 (m, 1H, CH_2_), 2.01 (m, 1H, CH_2_), 1.84 (m, 1H, CH_2_), 1.70–1.61 (m, 3H, CH_3_). ^13^C NMR (125.7 MHz, CDCl_3_, conformer mixture, signals of both *E *and *Z *isomer): *δ* = 169.6/169.4 (COO), 152.3/152.0 (NCOO), 134.9/134.9 (Ar-C), 134.6/134.5 (Ar-C), 130.0/129.9 (CH), 128.6/127.8 (10 Ar-CH), 123.2/122.8/122.1/122.0 (CH)_cis&trans_, 122.0/121.5/120.1/119.8 (CH)_cis&trans_, 116.0/115.2 (C), 67.0/66.9 (Bn-CH_2_), 65.9/65.9 (Bn-CH_2_), 52.8/52.4 (CH) 22.5/22.1 (CH_2_), 21.6/21.1 (CH_2_), 17.5/17.2 (CH_3_). C_24_H_25_NO_4_ (391.17). HRMS (EI) *m*/*z* = 391.17570; calcd. for [C_24_H_25_NO_4_]: *m*/*z* = 391.17586.

### 2.4. General Procedure for Wittig Reaction with Stable Ylids

The corresponding 2-(triphenylphosphoranylidene)acetate (0.067 mmol, 1.8 eq.) was added to the solution of protected aldehyde **6** (0.037 mmol, 1 eq., 100 mg) in dry toluene (7 ml), and the reaction mixture was refluxed for 18 h. The progress of the reaction was monitored by TLC till the aldehyde was consumed. After completion, the reaction mixture was concentrated and the product was purified chromatographically (pet. Ether/EtOAc 7 : 3) to afford the desired product.

#### 2.4.1. Dibenzyl (*R,E*)-5-(3-Ethoxy-3-oxo-1-propenyl)-3,4-dihydropyridine-1,2(2*H*)-dicarboxylate (**14a**)

Pale-yellow oil, yield 80 mg (67%). [*α*]_D_^23^ = +18.2 (*c* = 1.5, CHCl_3_); ^1^H NMR (500 MHz, CDCl_3_, conformer mixture): *δ* = 7.46–7.25 (m, 12H, Ar-H, CH), 5.68/5.67 (d, *J* = 15.5 Hz, 1 CH), 5.32–5.10 (m, 4H, Bn-CH_2_), 4.99 (m, 1H, CH), 4.24/4.22 (q, *J* = 6.8 Hz, 2H, CH_2_), 2.52–2.40 (m, 1H, CH_2_), 2.19 (m, 1H, CH_2_), 1.99–1.86 (m, 2H, CH_2_), 1.31 (t, *J* = 6.4 Hz, 3H, CH_3_). ^13^C NMR (125.7 MHz, CDCl_3_, conformer mixture): *δ* = 170.0/169.9 (COO), 167.3/167.3 (COOC_2_H_5_), 152.8/152.8 (NCOO), 145.1/145.0 (CH=CHCOO), 135.3/135.2 (2 Ar-C), 132.0/131.6 (CH=C), 128.7 (10 Ar-CH), 115.5/115.0 (C=CH), 113.6/113.5 (CHCOO), 68.7/68.5 (Bn-CH_2_), 67.3/67.2 (Bn-CH_2_), 60.0/59.9 (CH_2_), 54.3/53.9 (CH), 23.0/22.7 (CH_2_), 18.0/17.8 (CH_2_), 14.3/14.2 (CH_3_). C_26_H_27_NO_6_ (449.18). HRMS (EI): *m*/*z* = 449.18190; calcd. for C_26_H_27_NO_6_: *m*/*z* = 449.18384.

#### 2.4.2. Dibenzyl (*R,E*)-5-(3-Methoxy-3-oxo-1-propenyl)-3,4-dihydropyridine-1,2(2*H*)-dicarboxylate (**14b**)

Pale yellow oil, yield 122 mg (71%). [*α*]_D_^23^ = +16.7 (*c* = 1.5, CHCl_3_); ^1^H NMR (500 MHz, CDCl_3_, conformer mixture): *δ* = 7.36–7.15 (m, 12H, Ar-H, CH), 5.57/5.58 (d, *J* = 15.4 Hz, 1H, CH), 5.21–5.02 (m, 4H, Bn-CH_2_), 4.96–4.82 (m, 1H, CH), 3.67/3.65 (s, 3H, CH_3_), 2.42 (m, 1H, CH_2_), 2.12 (m, 1H, CH_2_), 1.89–1.76 (m, 2H, CH_2_). ^13^C NMR (125.7 MHz, CDCl_3_, conformer mixture): *δ* = 170.0/169.90 (COO), 167.8/167.8 (COOCH_3_), 152.8/152.8 (NCOO), 145.4 (CH=CHCOO), 135.3/135.2 (2 Ar-C), 132.2/131.8 (CH=C), 128.7 (10 Ar-CH), 115.4/115.0 (C=CH), 113.1/113.0 (CHCOO), 68.8/68.5 (Bn-CH_2_), 67.3/67.2 (Bn-CH_2_), 54.3/53.9 (CH), 51.7/51.1 (CH_3_), 22.9/22.7 (CH_2_), 18.0/17.8 (CH_2_). C_25_H_25_NO_6_ (435.16). HRMS (EI): *m*/*z* = 435.16780; calcd. for C_25_H_25_NO_6_: *m*/*z* = 435.16819.

#### 2.4.3. Dibenzyl (*R,E*)-5-(3-Benzyloxy-3-oxo-1-propenyl)-3,4-dihydropyridine-1,2(2*H*)-dicarboxylate (**14c**)

Colorless oil, yield 83 mg (62%). [*α*]_D_^23^ = +14.9 (*c* = 1.5, CHCl_3_); ^1^H NMR (500 MHz, CDCl_3_, conformer mixture): *δ* = 7.49–7.27 (m, 17H, Ar-H, CH), 5.75/5.76 (d, *J* = 14.6 Hz, 1H, CH), 5.77–5.12 (m, 6H, Bn-CH_2_), 4.98 (m, 1H, CH), 2.51 (m, 1H, CH_2_), 2.22 (m, 1H, CH_2_), 2.00–1.88 (m, 2H, CH_2_). ^13^C NMR (125.7 MHz, CDCl_3_, conformer mixture): *δ* = 170.0/169.9 (COO), 167.2/167.1 (COOCH_2_C_6_H_5_), 152.8/152.8 (NCOO), 145.7/145.7 (CH=CHCOO), 136.4/136.3 (2 Ar-C), 135.3/135.2 (Ar-C), 132.4/132.0 (CH=C), 128.7 (15 Ar-CH), 115.5/115.0 (C=CH), 113.2/113.1 (CHCOO), 68.8/68.5 (Bn-CH_2_), 67.3/67.3 (Bn-CH_2_), 66.0/65.9 (Bn-CH_2_), 54.3/53.9 (CH), 23.0/22.8 (CH_2_), 18.1/17.8 (CH_2_). C_31_H_29_NO_6_ (511.20). HRMS (EI): *m*/*z* = 511.20030; calcd. for C_31_H_29_NO_6_: *m*/*z* = 511.19949.

### 2.5. General Procedure for the Preparation of Enamino Esters **17**

A solution of lithium diisopropylamide (0.391 mmol, 2 M in heptanes) in THF (5 mL) was cooled to −78°C. Substituted acetate (0.778 mmol, 1 eq.) in THF (1 mL) was added dropwise over 30 min, and the solution was allowed to stir for a further 30 min. Protected lactam **16** (0.778 mmol, 1 eq.) in THF (2 mL) was added over 30 min at −78°C. Then the solution was allowed to warm up to RT and stirred for 18 h. Satd. aq. NH_4_Cl solution (10 mL) was added and the mixture was extracted with CH_2_Cl_2_ (3 × 100 mL). The combined organic phase was washed with brine (2 × 150 mL) and dried over MgSO_4_, and the solvent was removed in vacuo. TFA (2.1 mL, 28 mmol) was added to the crude residue, and the resulting mixture was stirred for 3 h at 25°C. Excess TFA was removed in vacuo and the resulting oil was dissolved in CH_2_Cl_2_ (5 mL). Sat. aq Na_2_CO_3_ was added to neutralize the solution, and the organic components were extracted with CH_2_Cl_2_ (3 × 15 mL). The combined organic phase was dried over MgSO_4_, and the solvent was evaporated in vacuo. The resulting yellow oil was purified by flash column chromatography (n-pentane/EtOAc 4 : 1) to give the enamino ester as pale-yellow oil.

#### 2.5.1. Methyl (*R,Z*)-6-(2-Methoxy-2-oxoethylidene)piperidine-2-carboxylate (**17a**)

Yellow oil, yield 55 mg (67%). [*α*]_D_^23^ = +15.7 (*c* = 1.5, CHCl_3_); ^1^H NMR (500 MHz, CDCl_3_, conformer mixture): *δ* = 9.01 (br s, 1H, NH), 4.48 (br s, 1H, C=CH), 4.05 (dd, *J* = 8.0 Hz, 5.5 Hz, 1H, CH), 3.78 (s, 3H, COOCH_3_), 3.63 (s, 3H, COOCH_3_), 2.39–2.36 (m, 2H, CH_2_), 2.19 (m, 1H, CH_2_), 1.90 (m, 1H, CH_2_), 1.79 (m, 1H, CH_2_), 1.68 (m, 1H, CH_2_). ^13^C NMR (125 MHz, CDCl_3_): *δ* = 172.2 (COO), 170.6 (COO), 160.8 (C=CH), 82.7 (C=CH), 53.6 (CH), 52.5 (CH_3_), 50.0 (CH_3_), 28.7 (CH_2_), 25.9 (CH_2_), 18.7 (CH_2_). C_10_H_15_NO_4_ (213.10). HRMS (EI) *m*/*z* = 213.09978; calcd. for [C_12_H_19_NO_4_]^+^: *m*/*z* = 213.09956.

#### 2.5.2. Methyl (*R,Z*)-6-(2-Ethoxy-2-oxoethylidene)piperidine-2-carboxylate (**17b**)

Yellow oil, yield 50 mg (56%). [*α*]_D_^23^ = +14.5 (*c* = 1.5, CHCl_3_); ^1^H NMR (500 MHz, CDCl_3_, conformer mixture): *δ* = 9.01 (br s, 1H, NH), 4.48 (br s, 1H, C=CH), 4.10 (q, *J* = 7.1 Hz, 2H, CH_2_), 4.04 (dd, *J* = 8.0 Hz, 5.5 Hz, 1H, CH), 3.77 (s, 3H, CH_3_), 2.37–2.34 (m, 2H, CH_2_), 2.19 (m, 1H, CH_2_), 1.89 (m, 1H, CH_2_), 1.77 (m, 1H, CH_2_), 1.64 (m, 1H, CH_2_), 1.25 (t, *J* = 7.1 Hz, 3H, CH_3_). ^13^C NMR (125 MHz, CDCl_3_): *δ* = 172.2 (COO), 170.3 (COO), 160.6 (C=CH), 82.7 (C=CH), 58.4 (CH_2_), 53.6 (CH_3_), 52.5 (CH), 28.8 (CH_2_), 25.9 (CH_2_), 18.8 (CH_2_), 14.6 (CH_3_). C_11_H_17_NO_4_ (227.12). HRMS (EI) *m*/*z* = 227.11548; calcd. for [C_11_H_17_NO_4_]^+^: *m*/*z* = 227.11521.

#### 2.5.3. Methyl (*R,Z*)-6-(2-*tert*-Butoxy-2-oxoethylidene)piperidine-2-carboxylate (**17c**)

Yellow oil, yield 65 mg (66%). [*α*]_D_^23^ = +12.9 (*c* = 1.5, CHCl_3_); ^1^H NMR (500 MHz, CDCl_3_, conformer mixture): *δ* = 8.92 (br s, 1H, NH), 4.43 (br s, 1H, C=CH), 4.05 (m, 1H, CH), 3.79 (s, 3H, COOCH_3_), 2.36–2.32 (m, 2H, CH_2_), 2.12 (m, 1H, CH_2_), 1.87 (m, 1H, CH_2_), 1.73 (m, 1H, CH_2_), 1.65 (m, 1H, CH_2_), 1.48 (s, 9H, C(CH_3_)_3_). ^13^C NMR (125 MHz, CDCl_3_): *δ* = 172.5 (COO), 170.3 (COO), 159.8 (C=CH), 84.7 (C=CH), 78.1 (C(CH_3_)_3_), 53.7 (CH_3_), 52.5 (CH), 28.8 (CH_2_), 28.6 (3 C(CH_3_)_3_), 26.1 (CH_2_), and 18.9 (CH_2_). C_13_H_21_NO_4_ (255.15). HRMS (EI) *m*/*z* = 255.14763; calcd. for [C_13_H_21_NO_4_]^+^: *m*/*z* = 255.14651.

#### 2.5.4. Ethyl (*R,Z*)-6-(2-Methoxy-2-oxoethylidene)piperidine-2-carboxylate (**17d**)

Yellow oil, yield 55 mg (66%). [*α*]_D_^23^ = +13.1 (*c* = 1.5, CHCl_3_); ^1^H NMR (500 MHz, CDCl_3_, conformer mixture): *δ* = 9.04 (br s, 1H, NH), 4.50 (br s, 1H, C=CH), 4.13 (q, *J* = 7.1 Hz, 2H, CH_2_), 4.07 (dd, *J* = 8.0 Hz, 5.5 Hz, 1H, CH), 3.80 (s, 3H, CH_3_), 2.35–2.32 (m, 2H, CH_2_), 2.16 (m, 1H, CH_2_), 1.88 (m, 1H, CH_2_), 1.75 (m, 1H, CH_2_), 1.67 (m, 1H, CH_2_), 1.27/1.26 (t, *J* = 7.1 Hz, 3H, CH_3_). ^13^C NMR (125 MHz, CDCl_3_): *δ* = 171.8 (COO), 170.6 (COO), 160.9 (C=CH), 82.2 (C=CH), 61.5 (CH_2_), 53.7 (CH), 50.0 (CH_3_), 28.8 (CH_2_), 25.9 (CH_2_), 18.7 (CH_2_), 14.1 (CH_3_). C_11_H_17_NO_4_ (227.12). HRMS (EI) *m*/*z* = 227.11521; calcd. for [C_11_H_17_NO_4_]: *m*/*z* = 227.11548.

#### 2.5.5. Ethyl (*R,Z*)-6-(2-Ethoxy-2-oxoethylidene)piperidine-2-carboxylate (**17e**)

Yellow oil, yield 47 mg (53%). [*α*]_D_^23^ = +19.6 (*c* = 1.5, CHCl_3_); ^1^H NMR (500 MHz, CDCl_3_, conformer mixture): *δ* = 9.01 (br s, 1H, NH), 4.48 (br s, 1H, C=CH), 4.24 (q, *J* = 7.1 Hz, 2H, CH_2_), 4.12 (q, *J* = 7.1 Hz, 2H, CH_2_), 4.03 (dd, *J* = 8.0 Hz, 5.4 Hz, 1H, CH), 2.36–2.32 (m, 2H, CH_2_), 2.15 (m, 1H, CH_2_), 1.84 (m, 1H, CH_2_), 1.75 (m, 1H, CH_2_), 1.67 (m, 1H, CH_2_), 1.30 (t, *J* = 7.1 Hz, 3H, CH_3_), 1.26 (t, *J* = 7.1 Hz, 3H, CH_3_). ^13^C NMR (125 MHz, CDCl_3_): *δ* = 170.8 (COO), 170.3 (COO), 160.7 (C=CH), 82.7 (C=CH), 61.5 (CH_2_), 58.4 (CH), 53.7 (CH_2_), 28.8 (CH_2_), 26.0 (CH_2_), 18.8 (CH_2_), 14.6 (CH_3_), 14.1 (CH_3_). HRMS (EI) *m*/*z* = 241.13120; calcd. for [C_12_H_19_NO_4_]: *m*/*z* = 241.13141.

## 3. Results and Discussion

**Scheme 1:**
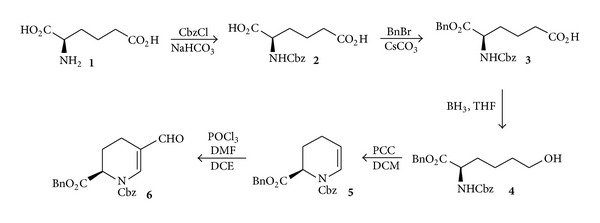
Synthesis of starting materials (alcohol **4**, enamine **5**, and aldehyde **6**) [10, 11].

We previously disclosed the syntheses of alcohol **4**, enamine **5**, and aldehyde **6** from (*R*)-*α*-aminoadipic acid (Scheme 1) [10, 11].

Amino acids with azido functions in the side chain are appreciated in organic synthesis for its ease of introduction into complex structures, convenient conversion to a primary amine [22], and participation in dipolar cycloaddition reactions [23], especially with respect to bioorthogonal reactions [24]. For the synthesis of the azide **8**, alcohol **4** was first converted into the methane sulfonate **7** using methanesulfonyl chloride and triethylamine. It was then converted into azide **8** by treatment with sodium azide in DMF at room temperature. 

**Scheme 2:**
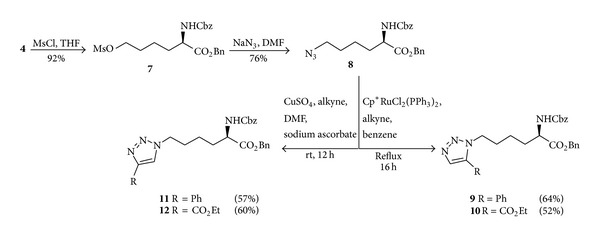
Synthesis of azide and triazole derivatives of (*R*)-*α*-aminoadipic acid.

Keeping in view the importance of triazole system [25] and with this azide intermediate in our hands, we performed click reactions using CuSO_4_/ascorbate and Cp*Ru(PPh_3_)_2_Cl_2_ as catalysts for the synthesis of 1,4- and 1,5-disubstituted triazole amino acid derivatives. Treatment of azide **8** with alkynes proceeded smoothly in the presence of a catalytic amount of CuSO_4_ and sodium ascorbate in a 10% solution of DMSO in water to produce 1,4-disubstituted-1,2,3-triazole derivatives **11** and **12** in good yields, respectively. The synthesis of 1,5-disubstituted triazole regioisomers was carried out successfully under ruthenium catalysis in good yields by refluxing azide **8** and catalyst and alkyne in benzene (Scheme 2). 

**Scheme 3:**
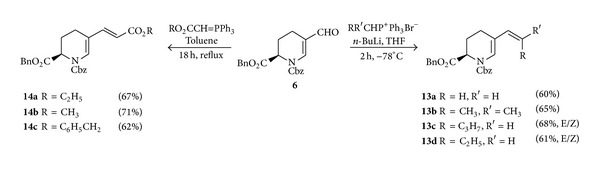
Synthesis of C-5-alkenyl derivatives of (*R*)-pipecolic acid.

Aldehyde moieties present in amino acids constitute a class of chiral synthons, valuable for the synthesis of optically active compounds and, in particular, for the synthesis of unusual amino acids. Thus, for the synthesis of C-5-alkenyl derivatives of (*R*)-pipecolic acid, formylation of enamine **5** by Vilsmeier-Haack reaction under reflux conditions was performed to provide protected aldehyde **6** (Scheme 1). One example of a Wittig reaction of aldehyde **6** giving a vinyl derivative of pipecolic acid has already been described by us [12]. In order to explore the scope of the reaction, aldehyde **6** was treated with a range of Wittig reagents to afford C-5-alkenyl derivatives of (*R*)-pipecolic acid. The reaction was carried out with nonstabilized ylids generated in situ from alkyl triphenylphosphonium bromides with *n*-BuLi at −78°C to afford the alkenyl substituted products in good yield. Compounds **13c** and **13d** were obtained as a mixture of E/Z isomers (Scheme 3; **13a–d**).

Wittig reaction of aldehyde **6** was also investigated with an array of stabilized ylids that are preformed and added to suitable carbonyl compounds. This reaction was performed in toluene as a solvent under refluxing conditions for 18 hours and provided exclusively *E*-configured *α*,*β*-unsaturated ester derivatives of pipecolic acid **14** (Scheme 3, **14a–c**).

Strongly acidic or basic reaction conditions often lead to epimerization or racemization in susceptible chiral compounds. Therefore, the enantiomeric purity of the reaction products **6** and **13a** was determined by chiral HPLC (Chiralpak-AD).

**Figure 1:**
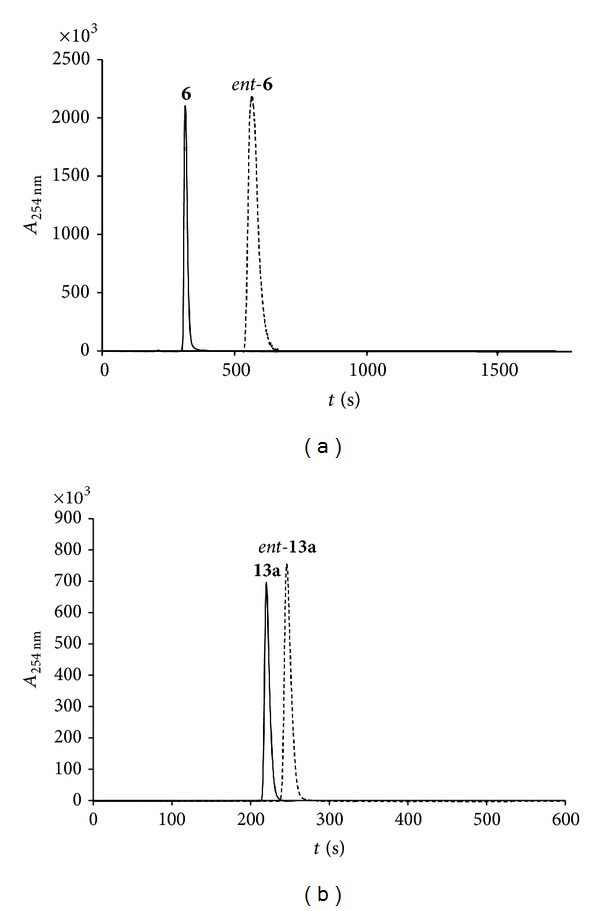
(a) Determination of enantiomeric purities by chiral HPLC analysis of **6**. **6** *t*_*R*_ = 5.26 min, *ent*-**6** *t*_*R*_ = 9.48 min. (b) Determination of enantiomeric purities by chiral HPLC analysis of **13a. 13a** *t*_*R*_ = 3.67 min, *ent*-**13a** *t*_*R*_ = 4.20 min.

**Scheme 4:**
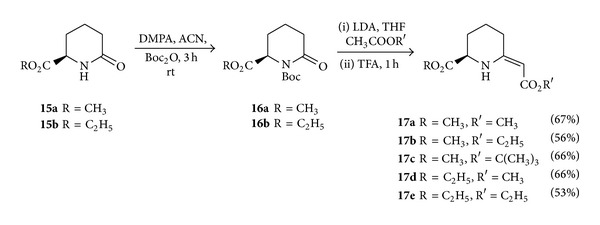
Synthesis of C-6 enamino esters of (*R*)-pipecolic acid.

For comparison the *S*-configured enantiomers *ent* **-6** and *ent* **-13a** were synthesized starting from (*S*)-*α*-aminoadipic acid and analyzed as well. No epimerization could be detected for the two compounds investigated (Figure 1).

(*R*)-6-Oxopipecolic acid derivatives **15 **can be easily prepared from (*R*)-*α*-aminoadipic acid in consecutive steps of esterification and cyclization during Kugelrohr distillation according to previously reported procedures [12, 26]. The amide group of **15** was Boc-protected with Boc anhydride and DMAP in acetonitrile at room temperature. For synthesis of C6-exocyclic enamino esters of pipecolic acid, compound **16** was reacted with a variety of substituted alkyl acetates in presence of LDA followed by treatment with TFA for 2 h at room temperature to give the desired compounds (Scheme 4). The *Z* configuration of the synthesized compounds was established on the basis of chemical shift values of the alkenyl and the NH protons. The chemical shift values for these protons are significantly deshielded which indicates the *Z* configuration, and this is in good agreement with the data reported for 5-membered cyclic analogs of these compounds [27].

In summary, efficient and simple syntheses of C-5 alkenyl and C-6 enamino ester derivatives of pipecolic acid are presented from enantiopure (*R*)-*α*-aminoadipic acid. Furthermore, the 6-azido and regioisomeric 6-triazolyl derivatives of amino (*R*)-*α*-aminohexanoic acid were prepared starting from (*R*)-*α*-aminoadipic acid by CuAAC or RuAAC.

## References

[1] Sardina JF, Rapoport H (1996). Enantiospecific synthesis of heterocycles from *α*-amino acids. *Chemical Reviews*.

[2] Rutjes FPJT, Wolf LB, Schoemaker HE (2000). Applications of aliphatic unsaturated non-proteinogenic *α*-H-*α*-amino acids. *Journal of the Chemical Society, Perkin Transactions 1*.

[3] Kaiser J, Kinderman SS, van Esseveldt BCJ (2005). Synthetic applications of aliphatic unsaturated *α*-H-*α*-amino acid. *Organic and Biomolecular Chemistry*.

[4] Gracia SR, Gaus K, Sewald N (2009). Synthesis of chemically modified bioactive peptides: recent advances, challenges and developments for medicinal chemistry. *Future Medicinal Chemistry*.

[5] Zhu X, Tang G, Galili G (2000). The catabolic function of the *α*-aminoadipic acid pathway in plants is associated with unidirectional activity of lysine-oxoglutarate reductase, but not saccharopine dehydrogenase. *Biochemical Journal*.

[6] McLennan H, Hicks TP, Liu JR (1982). On the configuration of the receptors for excitatory amino acids. *Neuropharmacology*.

[7] Olney JW, Labruyere J, Collins JF, Curry K (1981). D-Aminaphosphonovalerate is 100-fold more powerful than D-*α*-aminoadipate in blocking N-methylaspartate neurotoxicity. *Brain Research*.

[8] Bergmeier SC, Cobls AA, Rapoport H (1993). Chirospecific synthesis of (1S,3R)-1-amino-3-(hydroxymethyl)cyclopentane, precursor for carbocyclic nucleoside synthesis. Dieckmann cyclization with an *α*-amino acid. *Journal of Organic Chemistry*.

[9] Sonawane VC (2006). Enzymatic modifications of cephalosporins by cephalosporin acylase and other enzymes. *Critical Reviews in Biotechnology*.

[10] Sadiq A, Sewald N (2012). (R)-*α*-aminoadipic acid: an interesting chiral pool building block. *Arkivoc*.

[11] Sadiq A, Sewald N (2013). 6-Alkynyl- and 6-aryl-substituted (R)-pipecolic acid derivatives. *Organic Letters*.

[12] Copeland TD, Wondrak EM, Tozser J, Roberts MM, Oroszlan S (1990). Substitution of proline with pipecolic acid at the scissile bond converts a peptide substrate of HIV proteinase into a selective inhibitor. *Biochemical and Biophysical Research Communications*.

[13] Ekenstam TBA, Bovin C L-N-n-Propylpipecolic acid-2,6-xylidide.

[14] Kikumoto R, Tamao Y, Tezuka T (1984). Selective inhibition of thrombin by (2R,4R)-4-methyl-1-[N2-[1,2,3,4-tetrahydro-8-quinolinyl)sulfonyl]-L-arginyl]-2-piperidinecarboxylic acid. *Biochemistry*.

[15] Flynn AG, Giruox LE, Dage CR (1987). An acyl-iminium ion cyclization route to a novel conformationally restricted dipeptide mimic: applications to angiotensin-converting enzyme inhibition. *Journal of the American Chemical Society*.

[16] Gatto GJ, Boyne MT, Kelleher NL, Walsh CT (2006). Biosynthesis of pipecolic acid by RapL, a lysine cyclodeaminase encoded in the rapamycin gene cluster. *Journal of the American Chemical Society*.

[17] Nakatsuka M, Ragan JA, Sammakia T, Smith DB, Uehling DE, Schreiber SL (1990). Total synthesis of FK506 and an FKBP probe reagent, (C8,C9-13C2)-FK506. *Journal of the American Chemical Society*.

[18] Boger DL, Chen J, Saionz KW (1996). (-)-Sandramycin: total synthesis and characterization of DNA binding properties. *Journal of the American Chemical Society*.

[19] Germann UA, Shlyakhter D, Mason VS (1997). Cellular and biochemical characterization of VX-710 as a chemosensitizer: reversal of P-glycoprotein-mediated multidrug resistance in vitro. *Anti-Cancer Drugs*.

[20] Weigelt S, Huber T, Hofmann F (2012). Synthesis and conformational analysis of efrapeptins. *Chemistry*.

[21] Miller TA, Witter DJ, Belvedere S (2003). Histone deacetylase inhibitors. *Journal of Medicinal Chemistry*.

[22] Scriven E, Turnbull K (1988). Azides: their preparation and synthetic uses. *Chemical Reviews*.

[23] Katritzky AR, Zhang Y, Singh SK (2003). 1,2,3-Triazole formation under mild conditions via 1,3-dipolar cycloaddition of acetylenes with azides. *Heterocycles*.

[24] Angell YL, Burgess K (2007). Peptidomimetics via copper-catalyzed azide-alkyne cycloadditions. *Chemical Society Reviews*.

[25] Gajewski M, Seaver B, Esslinger SC (2007). Design, synthesis, and biological activity of novel triazole amino acids used to probe binding interactions between ligand and neutral amino acid transport protein SN1. *Bioorganic and Medicinal Chemistry Letters*.

[26] Huang SB, Nelson JS, Weller DD (1989). Preparation of optically pure *ω*-hydroxymethyl lactams. *Synthetic Communications*.

[27] Elliott MC, Wordingham SV (2006). A convenient protocol for the alkylidenation of lactams. *Synthesis*.

